# The Importance of Disease-specific Growth Charts for Children with Congenital Adrenal Hyperplasia

**DOI:** 10.1210/clinem/dgaf554

**Published:** 2025-10-06

**Authors:** Kyriakie Sarafoglou, Yesica Mercado Munoz, Charles Sukin, Aida Lteif, Jennifer Kyllo, Bradley S Miller, O Yaw Addo, Deborah P Merke

**Affiliations:** Division of Pediatric Endocrinology, University of Minnesota Medical School, Minneapolis, MN 55454, USA; Department of Experimental and Clinical Pharmacology, University of Minnesota College of Pharmacy, Minneapolis, MN 55455, USA; Division of Pediatric Endocrinology, University of Minnesota Medical School, Minneapolis, MN 55454, USA; Department of Pediatrics, National Institutes of Health Clinical Center, Bethesda, MD 20892, USA; Division of Pediatric Endocrinology and Metabolism, Department of Pediatric and Adolescent Medicine, Mayo Clinic, Rochester, MN 55905, USA; Division of Endocrinology, Children's Hospitals and Clinics of Minnesota, St. Paul, MN 55102, USA; Division of Pediatric Endocrinology, University of Minnesota Medical School, Minneapolis, MN 55454, USA; Rollins School of Public Health, Emory University, Atlanta, GA 30322, USA; Department of Pediatrics, National Institutes of Health Clinical Center, Bethesda, MD 20892, USA; *Eunice Kennedy Shriver* National Institute of Child Health and Human Development, Bethesda, MD 20892, USA

**Keywords:** congenital adrenal hyperplasia, growth, growth chart, 21-hydroxylase deficiency

## Abstract

**Background:**

Children with congenital adrenal hyperplasia (CAH) due to 21-hydroxylase deficiency typically have height, weight, and body mass index (BMI) growth patterns that differ from the general population due to increased androgen and/or glucocorticoid exposures. With the recent surge in the development of new therapies, CAH-specific growth charts are needed to evaluate the effectiveness of these new treatments.

**Methods:**

Retrospective data from patients aged 0 to 20 years with classic CAH, confirmed by hormonal testing and/or CYP21A2 genotyping, from 2 large clinical databases were analyzed. Specialized charts were developed using the lamda-mu-sigma semiparametric modeling method to generate CAH-specific percentile curves from 0 to 20 years. Nodal-point analyses were conducted to assess differences in incremental growth at 4, 8, 12, 16, and 20 years of age relative to Centers for Disease Control and Prevention (CDC) 2000 normative charts using 1-sided quantile tests and age of adiposity rebound estimated with curve derivative solutions.

**Results:**

A total sample of 8692 visits from 515 patients was used. Growth (height-, weight-, BMI-for-age) channels of CAH patients were significantly different over the entire growing period and characterized by diminished pubertal spurt relative to the CDC reference. Onset of adiposity rebound based on BMI-for-age occurred earlier for CAH patients (females 3.3 years, males 3.9 years) compared to their normative counterparts (5-8 years).

**Conclusion:**

Our study showed that at incremental time points throughout childhood, children with CAH collectively follow specific differences in growth trajectories as compared to unaffected children. These variations highlight the need for CAH-specific charts to assist in clinical management, appraisal of growth trajectories, and assessment of the impact of new therapies.

Classic congenital adrenal hyperplasia (CAH) due to 21-hydroxylase deficiency (21OHD) results from an enzymatic defect in the cortisol synthesis pathway and shifts hormone precursors to the androgen pathway, resulting in excessive production of adrenal androgens. Patients require lifelong treatment with glucocorticoids to address cortisol insufficiency but often at supraphysiological dosing to reduce excess adrenal androgens (1, 2). Because of the varying degrees of increased androgen and/or glucocorticoid exposures, children with CAH typically have physical growth patterns [height, weight, body mass index (BMI)] and pubertal development that differ from the general population (3-6).

The negative impact that both the disease and its treatment have on linear growth is well documented, with adult height often 1 to 2 SDs below target height and that of the control adult population (4, 7, 8). Intrinsic to the disease is the production of excess androgens, which, through aromatization into estrogens, lead to accelerated growth velocity, advanced skeletal maturation, and early growth plate fusion, leading to adult short stature. Chronic supraphysiological glucocorticoid dosing impairs linear growth by inhibiting chondrocyte proliferation at the growth plate, interfering with the production and secretion of GH, inhibiting GH production of insulin-like growth factor 1 messenger RNA in liver cells, and creating a state of GH resistance at the growth plate, also leading to adult short stature (9, 10).

Long-term glucocorticoid overexposure that begins early in life may result in increased weight gain, adiposity rebound, hypertension, and insulin resistance, all of which can increase the risk of metabolic syndrome and cardiovascular disease in adulthood. Multiple studies report that the rate of obesity (BMI ≥95th percentile) in youth with CAH exceeds that of obesity in the general population (20%) with most reporting rates of over 30% (11-17). However, it is important to consider that some of the higher prevalence of obesity seen in pediatric CAH patients may be due to misclassification. Early puberty, tall stature, and increased lean body mass due to increased androgen exposure can lead to a higher prevalence of elevated BMI that is misclassified as obesity (12).

As growth and weight patterns of children with CAH differ from the general population, there is a need for CAH-specific growth charts that can help guide treatment decisions and evaluate the effectiveness of new treatments in clinical trials. Therefore, our aim was to develop CAH-specific height-for-age, weight-for-age, and BMI-for-age reference growth charts for children ages birth through 20 years and to evaluate perturbations of growth and development in a large multicenter cohort of children with classic 21OHD.

## Materials and Methods

### Data Sources and Statistical Methods

Data used in this study came from 2 clinical cohorts. The first is the Minnesota Congenital Adrenal Hyperplasia Cohort (University of Minnesota Masonic Children's Hospital, Children’s Hospitals of Minnesota, and Mayo Clinic). The database with de-identified data was hosted at the University of Minnesota using the Research Electronic Data Capture tool and approved by the institutional review board at the University of Minnesota. The Research Electronic Data Capture tool is a secure, Health Insurance Portability and Accountability Act-protected, web-based software platform. The second is the National Institutes of Health (NIH) Clinical Center cohort, consisting of patients enrolled in the Natural History Study of Patients with Excess Androgens conducted at the NIH Clinical Center in Bethesda, Maryland (www.ClinicalTrials.gov identifier no. NCT00250159). The NIH institutional review board approved the study. All parents or guardians of participating children and adults (≥18 years of age) gave written informed consent, and all minors at least 8 years old gave assent.

For both cohorts, anthropometric data were collected using electronic medical records and paper-based charts up to age 20 retrospectively from patients with classic 21OHD [salt-wasting (SW) and simple virilizing (SV)]. Diagnosis was confirmed with hormonal testing and/or *CYP21A2* genotyping. Patients were excluded if there was no record of anthropometric data prior to 16 years of age. Bone age was determined using the Greulich and Pyle method by a pediatric endocrinologist starting at a chronological age of 4 years. A patient was determined to have reached adult height when bone age was 17 years or greater for males and 15 years or greater for females. Patients were on hydrocortisone (HC) and fludrocortisone during their growing years.

Cohort descriptive characteristics were presented as percentages or means/medians for categorical and continuous variables, respectively. Patient descriptive statistics were presented by counts and by clinic visits or instances at which growth data were measured.

### CAH-specific Growth Charts

The CAH growth charts were developed using the lamda-mu-sigma (LMS) modeling method. The generalized additive model for location scale and shape technique of the LMS method was used (18, 19). This approach uses complex distribution curve parameters and age-smoothed splines to characterize growth as a time (age) function, viz. height-for-age, weight-for-age, and BMI-for-age curves. The LMS is a well-known method for growth reference analyses, including those from the CDC and World Health Organization (18, 20, 21). The growth charts are displayed as percentiles along with LMS parameters needed for calculating CAH-specific *z*-scores. To select final models, extensive statistical and visual diagnostic tools were used (19, 22). The pediatric growth cleaner tool (23) was used to identify possible clerical errors in anthropometric data such as same-day measurements, duplicates, and last measurement carried forward before statistical modeling.

All participants in this cohort of patients with CAH received glucocorticoids, and the majority also received fludrocortisone as standard therapy. Additional therapies that some patients received were GH, GnRH agonist (GnRHa) therapy, and aromatase inhibitors. We ascertained if growth trajectories differed according to each additional therapy using visual exploratory data analysis techniques (24). Sensitivity analyses using scattergrams with smoothed running median plots by type of therapy (ever received) and stratified by patient sex for each anthropometric measure were done. No differences in growth patterns were observed. Growth-curve models using all pooled patient data without exclusions were completed.

To assess differences in overall growth patterns, the CAH-specific percentiles were overlaid on CDC 2000 growth charts from birth (0) to 36 months and ages 2 to 20 years old. Further, nodal-point analysis was conducted using 1-sided quantile tests for children 0 to 20 years at 4-year increments. CAH-specific growth patterns were illustrated by charting stature and weight parameters of patients with CAH on a CAH-specific growth chart along with the CDC reference growth chart as background.

We evaluated BMI classification of the CAH patient cohort according to CDC and CAH-specific BMI charts for the entire cohort at 4, 8, 12, 16, and 20 years of age. BMI percentiles curves for the CAH-specific growth charts were derived from BMI datapoints calculated with the available heights and weights collected at time of patient visit. BMI categories for overweight (85th-95th), obese (>95th) percentile, and severe obesity defined as 120th of the CDC 95th percentile of the CDC extended BMI-for-age chart were examined (25, 26). Restricted cubic spline modeling (27) with differential calculus (28) was used to calculate the timing of adiposity rebound based on smoothed median BMI nadir, which was estimated by sex and CAH subtype. After birth, BMI increases, peaking at around the first year of life. Afterward, BMI decreases gradually, reaching a nadir on average at around 5 to 6 years of age, at which point BMI increases through childhood to adulthood. The point at BMI nadir is defined as adiposity rebound. All statistical analyses and visualizations were conducted in R (The R Foundation for Statistical Programming) (28), and significance was set at a *P*-value of .05, where appropriate.

## Results

Data came from 515 (279 female) patients with CAH due to classic 21OHD; 67% were of the SW phenotype (Table 1). The median age at diagnosis was 0.1 to 0.2 months for patients with SW CAH. Female patients with SV CAH were diagnosed at a median age of 0.5 months and males at 48 months. A total sample of 8692 datapoints was used to develop the CAH-specific growth charts (8692 height, 8677 weight, and 8635 BMI) (Table 1). The average time of cohort follow-up was 8.1 years (SD 5.4) for females and 8.1 (SD 5.2) years for males with a range of 0 to 20 years. All patients were on HC, and the majority also received fludrocortisone therapy throughout, with some patients receiving add-on therapies. For example, while 63% of patients in this cohort were on standard therapy only, 13% received GnRHa, 10% received aromatase inhibitors, and 4% received GH at some point during their growing period. When examined by the number of clinic visits where growth measurements were obtained, about 17% of the visits were from patients receiving GnRHa, with ∼9% of those visits coming from patients receiving both GnRHa and an aromatase inhibitor.

**Table 1.** Characteristics of longitudinal cohort of patients with CAH aged 0 to 20 years

Characteristics	Patients (n = 515)	By number of visits with growth measurements (n = 8701)
Female, n (%)	279 (54.2)	52.4 (4557)
CAH subtype, n (%)		
Salt-wasting	344 (66.8)	68.9 (5998)
Simple virilizing	171 (33)	30.8 (2681)
Average age at diagnosis (months)	Median (IQR)	—
Salt-wasting—females (n = 189)	0.1 (0.0,0.2)	—
Salt-wasting—males (n = 155)	0.2 (0.1, 0.5)	—
Simple virilizing—females (n = 90)	0.5 (0.1, 13.2)	—
Simple virilizing—males (n = 81)	48.0 (1.4, 60.0)	—
Treatments,*^a^* n (%)		
Standard therapy (no add-on therapy)	396 (63.5)	5725 (47.9)
Standard + GH	26 (4.2)	686 (5.7)
Standard + GnRH analogue	80 (12.81)	1989 (16.6)
Standard + aromatase inhibitor	65 (10.4)	1822 (15.2)
Standard + GnRH analogue + aromatase inhibitor	34 (5.4)	1026 (8.6)
Standard + GH + GnRH analogue	15 (2.4)	441 (3.7)
Standard + GH + GnRH analogue + aromatase inhibitor	8 (1.3)	267 (2.2)
	**Anthropometric measurements according to visit instances**	
Measures used in CAH charts	Female	Male
Height from 8692visits	4548	4144
Weight from 8677 visits	4546	4131
BMI from 8635 visits	4529	4106

Abbreviations: BMI, body mass index; CAH, congenital adrenal hyperplasia; IQR, interquartile range.

^*a*^Standard therapy: hydrocortisone ± fludorocortisone only; add-on therapies based on ever exposed to any of the treatments over the growing period.

### CAH-specific Growth Patterns

**Figure 1.**
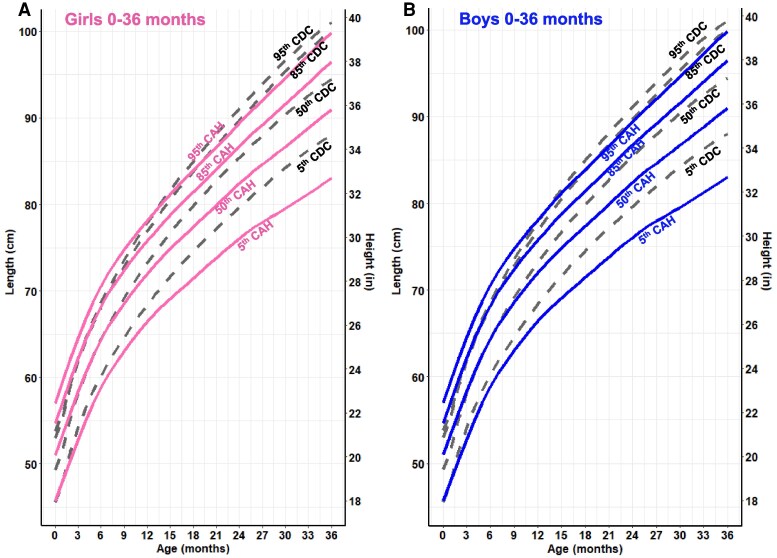
CAH-specific length-for-age growth curves for females (A) and males (B) ages 0 to 36 months overlaid on CDC 2000 reference growth curves. Solid blue lines represent males and solid pink lines represent females on the CAH curve. Dashed black line represents the CDC curve. Abbreviations: CAH, congenital adrenal hyperplasia; CDC, Centers for Disease Control and Prevention.

**Figure 2.**
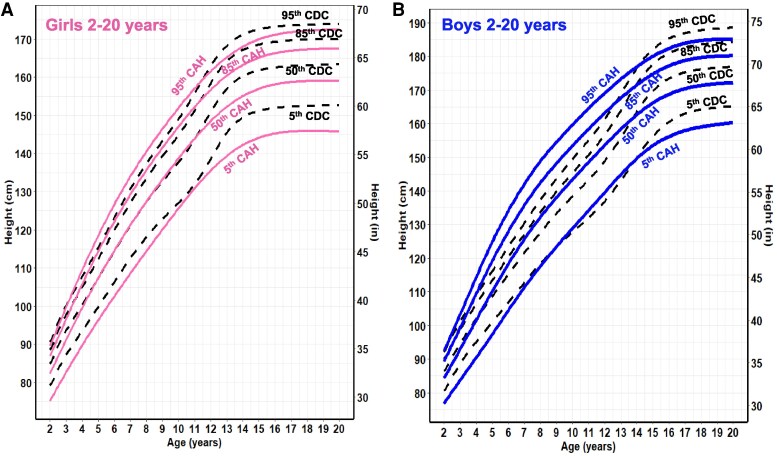
CAH-specific height-for-age growth curves for females (A) and males (B) ages 2 to 20 years overlaid on CDC 2000 reference growth curves. Solid blue lines represent males, and solid pink lines represent females on the CAH curve. Dashed black line represents the CDC curve. Abbreviations: CAH, congenital adrenal hyperplasia; CDC, Centers for Disease Control and Prevention.

**Figure 3.**
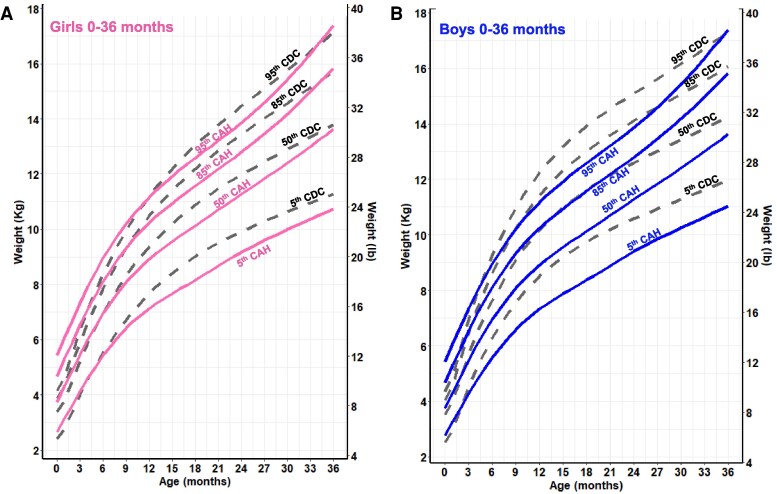
CAH-specific weight-for-age growth curves for females (A) and males (B) ages 0 to 36 months overlaid on CDC 2000 reference growth curves. Solid blue lines represent males, and solid pink lines represent females on the CAH curve. Dashed black line represents the CDC curve. Abbreviations: CAH, congenital adrenal hyperplasia; CDC, Centers for Disease Control and Prevention.

**Figure 4.**
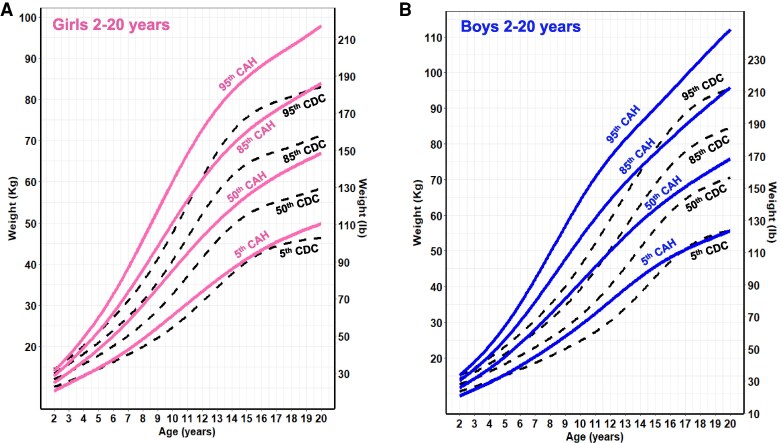
CAH-specific weight-for-age growth curves for females (A) and males (B) ages 2 to 20 years overlaid on CDC 2000 reference growth curves. Solid blue lines represent males, and solid pink lines represent females on the CAH curve. Dashed black line represents the CDC curve. Abbreviations: CAH, congenital adrenal hyperplasia; CDC, Centers for Disease Control and Prevention.

**Figure 5.**
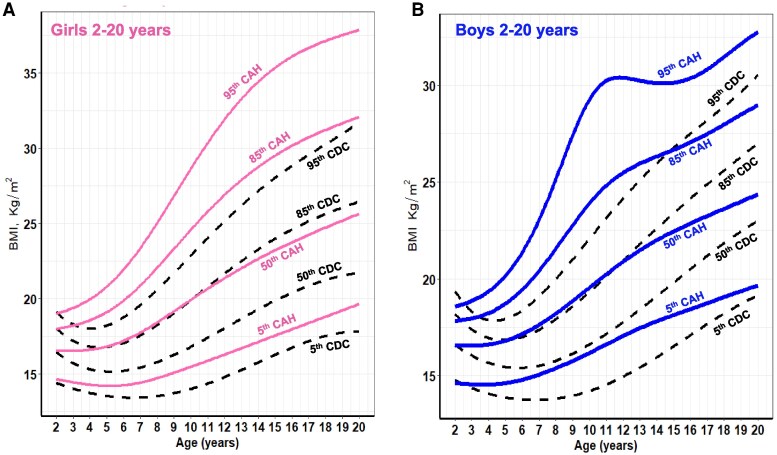
CAH-specific BMI-for-age growth curves for females (A) and males (B) ages 2 to 20 years overlaid on CDC 2000 reference growth curves. Solid blue lines represent males, and solid pink lines represent females on the CAH curve. Dashed black line represents the CDC curve. Abbreviations: BMI, body mass index; CAH, congenital adrenal hyperplasia; CDC, Centers for Disease Control and Prevention.

Overall, CAH growth patterns were markedly different from those of the CDC reference curves (Figs. 1-5). Nodal point analysis along each growth curve, relative to normative charts, revealed different growth patterns over time (Table 2). The height of CAH patients at 4 and 8 years was comparable to normative data at the median for females but not thereafter, such that CAH patients were 5 cm shorter than their normative data at 20 years (158 vs 163 cm in females, 171 vs 176 cm in males). Interestingly, boys with CAH were taller than the normative data at 8 and 12 years of age but then shorter at 16 and 20 years of age. In addition, the CAH-specific linear growth pattern showed a less pronounced pubertal growth spurt when visually comparing curve slopes.

**Table 2.** Classic CAH nodal point analysis relative to CDC 2000 normative charts by sex for children aged 0 to 20 years

	Median estimates—females					Median estimates—males				
	Height (cm)					Height (cm)				
Age (years)	CAH	CDC	*P*-diff*^a^*	CDC-SDS	CAH-SDS	CAH	CDC	*P*-diff*^a^*	CDC-SDS	CAH-SDS
4	103.5	103.9	.2095	−0.15	−0.03	106.0	105.3	.3961	0.17	0.02
8	130.5	130.6	.7411	0.02	−0.01	134.8	130.5	<.0001	0.71	−0.04
12	150.5	154.3	<.0001	−0.55	−0.02	156.0	152.1	<.0001	0.59	0.04
16	158.6	162.7	<.0001	−0.63	0.04	170.1	174.3	<.0001	−0.54	0.08
20	158.4	163.1	.2095	−0.71	−0.05	170.8	176.1	<.0001	−0.76	−0.09

	Weight (kg)					Weight (kg)				
Age (years)	CAH	CDC	*P*-diff*^a^*	CDC-SDS	CAH-SDS	CAH	CDC	*P*-diff*^a^*	CDC-SDS	CAH-SDS
4	17.7	16.7	<.0001	0.48	0.02	18.2	17.2	<.0001	0.52	−0.06
8	32.1	27.4	<.0001	0.82	−0.05	33.8	26.9	<.0001	1.20	−0.09
12	49	43.6	<.0001	0.55	0.06	54.2	42.8	<.0001	1.01	0.11
16	59.4	54.5	<.0001	0.49	−0.06	66.0	62.4	.0021	0.27	−0.05
20	63.1	56.2	<.0001	0.59	−0.07	68.6	67.3	.0333	0.13	−0.18

	BMI (kg/m^2^)					BMI (kg/m^2^)				
Age (years)	CAH	CDC	*P*-diff*^a^*	CDC-SDS	CAH-SDS	CAH	CDC	*P*-diff*^a^*	CDC-SDS	CAH-SDS
4	16.6	15.2	<.0001	0.94	−0.05	16.7	15.5	<.0001	0.89	0.01
8	18.7	16.1	<.0001	1.02	−0.07	18.5	15.9	<.0001	1.11	−0.03
12	21.3	18.4	<.0001	0.90	−0.06	21.4	18.1	<.0001	1.04	0.06
16	23.8	20.7	<.0001	0.79	−0.08	22.5	20.8	<.0001	0.56	−0.14
20	24.4	21.3	<.0001	0.80	−0.07	23.1	21.8	<.0001	0.4	−0.15

*P*-values estimated from 1-sample quantile test of differences between CDC chart 50th and CAH-specific median for each growth metric at ages 4, 8, 12, 16, and 20 years.

Abbreviations: CAH, congenital adrenal hyperplasia; CDC, Centers for Disease Control and Prevention; SDS, SD score.

^*a*^ *P*-values estimated from unsmoothed CAH cohort data.

**Figure 6.**
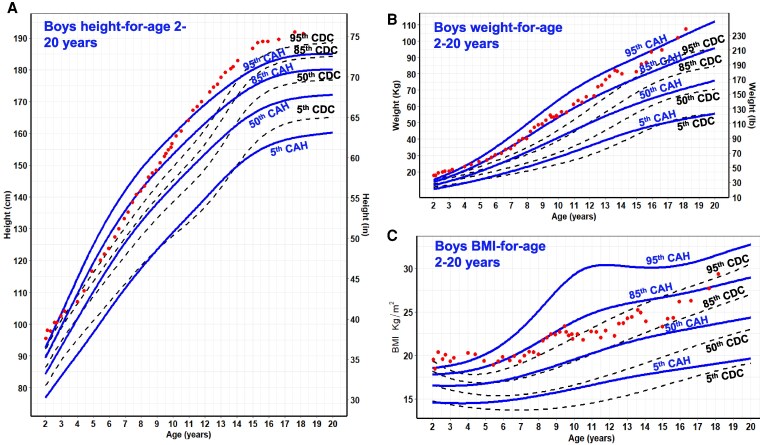
The height (A), weight (B), and BMI-for-age (C) of a representative male with simple virilizing CAH plotted in red dots on CAH-specific and CDC 2000 normative charts. He was diagnosed at 3.9 years, when he presented with a bone age of 9 years. Hydrocortisone therapy was initiated at the time of diagnosis. GH therapy and anastrozole were also started at 4.2 years and GnRHa at 9.5 years. GH and GnRHa were discontinued at 13.75 years. Anastrozole was stopped 1 month later. In the CDC BMI chart, he was classified as obese (>95th percentile) throughout his life. In contrast, on the CAH-specific BMI chart, he was classified as obese up to age 5 years, overweight (85th-95th percentile) from age 5 to 6 years of age, and under the 85th percentile until age 18 due to his tall stature and increased lean body mass as measured by DXA, which ranged from 52% to 84%. Solid blue lines represent males on the CAH curve. Dashed black line represents the CDC curve. Abbreviations: BMI, body mass index; CAH, congenital adrenal hyperplasia; CDC, Centers for Disease Control and Prevention; DXA, dual-energy X-ray absorptiometry; GnRHa, GnRH agonist.

For weight and BMI, CAH-specific percentile scores were above/higher at each corresponding percentile on the CDC chart and at each nodal point. For example, girls with CAH weighed on average 5 kg more at every age beginning at 8 years, and these differences were even more pronounced in boys. In both males and females, the 50th CAH-specific BMI-for-age percentile tracked above the CDC BMI 85th percentile from 2 to 12 years, suggesting that patients with CAH would be classified as overweight during these periods. Similarly, the CAH-specific percentile is higher at the 95th percentile. The importance of classifying overweight/obesity status in children with CAH using the CAH- specific charts is shown in Fig. 6 by a representative male with SV CAH who presented at 3 years and 11 months with tall stature and a bone age of 9 years. Although he was classified as obese (>95th percentile) throughout his life when using the CDC BMI chart, he was classified as obese up to age 5 years, overweight from age 5 to 6 years, and under the 85th percentile until age 18 years when using the CAH-specific BMI chart.

**Figure 7.**
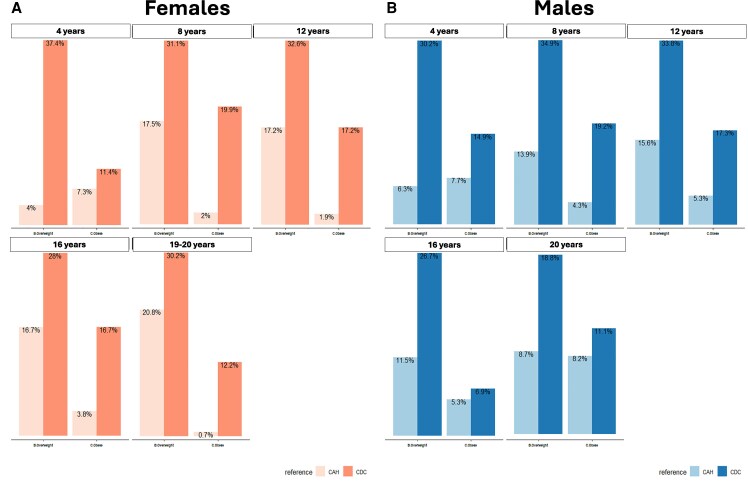
Differences in BMI classifications of overweight and obese females (A) and males (B) at ages 4, 12, 16, and 20 years of the CAH cohort according to the CAH-specific reference charts and the CDC reference charts. Overweight, 85thto 95th percentile; obese, >95th percentile. Abbreviations: BMI, body mass index; CAH, congenital adrenal hyperplasia; CDC, Centers for Disease Control and Prevention.

**Figure 8.**
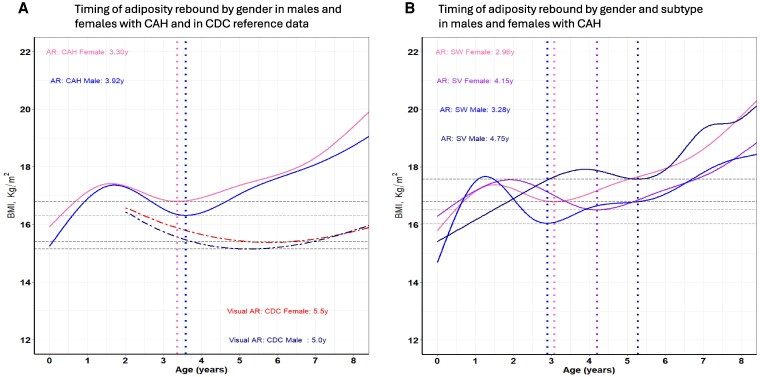
Timing of adiposity rebound by sex and subtype. Panel A shows the timing of adiposity rebound in males and females with CAH and in CDC reference data. CAH males, solid blue line; CAH females, solid pink line; CDC males, dark blue dashed line; CDC females, red dashed line; dotted vertical lines in blue show timing of adiposity rebound in males with CAH and in pink timing of adiposity rebound in females with CAH. Panel B shows the timing of adiposity rebound by sex and subtype in males and females with CAH. salt-wasting CAH males, solid blue line; salt-wasting CAH female, solid pink line; simple virilizing CAH male, solid dark blue line; simple virilizing CAH female, solid purple line. Dotted vertical line in blue shows timing of adiposity rebound in males with salt-wasting CAH; dotted vertical line in pink shows timing of adiposity rebound in females with salt-wasting CAH; dotted vertical line in dark blue shows timing of adiposity rebound in males with simple virilizing CAH; dotted vertical line in purple shows timing of adiposity rebound in females with simple virilizing CAH. Abbreviations: CAH, congenital adrenal hyperplasia; CDC, Centers for Disease Control and Prevention.

Differences in BMI classifications of overweight (85th-95th percentile), obese (>95th percentile), and severe obese (at ages 4, 12, 16, and 20 years of the CAH cohort according to the CAH-specific reference charts and the CDC extended BMI reference charts (25, 26) revealed marked differences (Fig. 7). Across all ages, sex, and BMI categories, the CDC charts classified children with CAH as overweight/obese multiple folds. While 4% of CAH cohort females were overweight at 4 years based on the CAH-specific chart, 37% were overweight based on the CDC BMI chart (∼9 times higher). Males were 7.7% overweight at 4 years based on the CAH-specific chart compared to 30% based on the CDC chart (∼4 times) higher. Severe obesity, as defined by the CDC and based on the extended BMI charts (25), had a prevalence range of 4.2% to 10.3% for females and males, respectively, over the life course of the cohort (data not shown). Timing of adiposity rebound for CAH females occurred at 3.30 years and 3.92 years for males compared to the CDC BMI curves for healthy females and males (5-6 years by visual inspection). Separated by sex and subtype, the adiposity rebound of SW females was 2.96 years, SW males 3.28 years, SV females 4.15 years, and SV males 4.75 years (Fig. 8). The differences between SW females vs SV females, SW females vs SW males, SV females vs SV males, and SW males vs SV males were all significant (*P* < .001).

## Discussion

Using data from 2 large cohorts of patients with CAH due to classic 21OHD, we created age- and sex-adjusted CAH-specific growth reference charts that show children with classic CAH have distinct growth and weight patterns when compared to the growth patterns of the general US population displayed in the CDC growth charts. The CAH-specific growth charts depict these differences at key stages of growth and development and provide a way to track these differences in relation to the CDC reference growth charts. It is especially important as new therapies become available for providers to have a way to evaluate these new treatment options specifically in relation to growth attainment expectations at defined nodal points of classic CAH patients (see Table 2).

Both males and females with classic CAH had a pronounced decreased pubertal height gain compared to the CDC reference population (2-20 years), consistent with other studies (3, 4). The decreased pubertal growth could be due to lower peak height velocity (3, 29) reflecting the impact of glucocorticoid suppression on linear growth and/or the impact of accelerated adrenal androgen-driven growth with early epiphyseal closure during the prepubertal years. Also noted during the first 3 years of life, children with classic CAH have decreased linear growth compared to the CDC reference data, reflecting the potential growth-suppressive effect of glucocorticoid therapy during this time period (30-32). Bomberg et al (3) postulated that patients with SV fared similarly or better than their SW counterparts in terms of adult height, despite their later age of diagnosis and advanced bone age, because of androgen-driven growth and a less prolonged exposure to glucocorticoids in the prepubertal time frame. Moreover, the growth-suppressive effect of supraphysiological doses of glucocorticoid therapy (doses greater than 15-20 mg/m^2^/day) has been described during 2 vulnerable developmental periods, infancy and puberty, in retrospective cohort studies.

It is well reported that adult height is reduced in children with classic CAH (4, 7, 8), reflecting the shortcomings of current therapy and the adverse effects of cumulative glucocorticoid and androgen exposure on linear growth, puberty development, and progression. The HC effect on predicted adult height was first quantified by Sarafoglou et al, who showed that each mg/m^2^/day increase in the average HC dose during the growth period was associated with a 0.37 cm average decrease in the predicted adult height in children with CAH. Pijnenburg-Kleizen et al found that each additional mg/m^2^/day of HC was associated with a reduction in near adult height of 0.13 SD score, which is equivalent to 0.78 cm in females and 0.89 cm in males (33).

With few exceptions (34, 35), it is also well reported that the rate of obesity in youth with CAH exceeds that of the general population (11-17, 36, 37). One potential cause, and a key difference in BMI accretion seen between the CAH-specific and CDC growth charts, is early adiposity rebound. In our study, we were able to derive that the time of onset of the CAH-specific BMI nadir occurs at 3.30 years for females and 3.92 years for males, earlier than the CDC BMI reference data for healthy females and males. This timing also exhibited sexual dimorphism by CAH subtype, with the adiposity rebound of SW and SV females occurring earlier than SW and SV males (Fig. 8). Earlier age at adiposity rebound in healthy controls has been associated with increased BMI during childhood and risk for metabolic syndrome (38), as well as increased adult obesity independent of parent obesity status and the BMI at adiposity rebound (39). In a retrospective study of 42 children with classic CAH, early adiposity rebound was associated with higher BMI at 7 and 12 years of age and central obesity in adolescence (40). A longitudinal study of metabolic risk factors in a cohort of patients with CAH that spanned childhood and adulthood showed that metabolic morbidity started prior to puberty, supporting the notion that metabolic risk begins early in life in patients with CAH (14).

With most cases of classic CAH identified at birth by newborn screening, treatment with higher doses of glucocorticoids during the first few years of life provides a possible explanation for early adiposity rebound as overtreatment was the common denominator in 3 studies that showed adiposity rebound in children with CAH occurs earlier, especially in the SW form (12, 41, 42). Recent data from the German/Austrian registry showed that the mean (lower-upper quartiles) daily HC dose (in mg/m^2^ of body surface area) for children with CAH was consistently at the high end of the recommended dosing range, with the youngest patients receiving HC doses as high as 19.8 mg/m^2^/day (43). Even though the glucocorticoid exposure over the last 2 decades has decreased, it is still supraphysiologic due to limitations of HC tablets that do not allow small incremental and precise dosing as the lowest dose is 2.5 mg from splitting a scored 5 mg tablet. However, new HC formulations such as Alkindi and the recently approved HC solution Khindivi (approved for children ≥5 years) allow for incremental dose changes as small as 0.1 to 0.5 mg. Small incremental doses over the first years of life may change the time of adiposity rebound in children with CAH. The CAH growth charts would capture these changes in therapy and their impact on adiposity rebound timing. Lastly, our finding of earlier adiposity rebound timing in both females and males with SW compared to their SV counterparts suggests that early adiposity rebound is not only dependent on the dose but also on the duration and cumulative exposure of glucocorticoids during the first 5 years of life. Children with SV missed by newborn screening and diagnosed at a later age with virilization, accelerated growth, and advanced bone age tend to be taller and have increased lean mass, contributing to higher BMI-for-age. Their later timing of adiposity rebound further underscores that prolonged glucocorticoid exposure predominates over the effect of androgen exposure on the timing of adiposity rebound.

Accelerated growth in children with CAH can lead to erroneous body composition estimates when using BMI-for-chronological-age (12, 44, 45). In a large cohort of healthy children, BMI-for-chronological-age clearly differed according to stature and was systemically higher in tall children compared to children with average height (45). We saw a similar pattern across all ages, sexes, and BMI categories as the CDC BMI charts classified a higher percentage of children with classic CAH as being overweight and obese compared to the CAH-specific BMI chart. The overestimation, especially during prepubertal years, can be due to excess adrenal androgen-driven growth or perhaps a later diagnosis that is seen in children with SV CAH. Overestimation of BMI in the CDC chart can be driven by tall stature due to growth acceleration and advanced bone age, even in the presence of increased lean body mass.

Our study has several limitations. Our patients were from multiple centers and received various treatment regimens, leading to heterogeneity in our study population. An additional limitation is the potential lack of precision in height and weight measurements, which were done at multiple clinics, although all were done in a pediatric endocrine clinic using a stadiometer. Despite these limitations, our disease-specific growth charts provide insight into the growth and development challenges experienced by children with classic CAH. A strength of the study is that the use of data from multiple centers that receive referrals from across the United States enlarged the cohort and makes our data more representative of the population used to generate the CDC reference curves.

Difficulty achieving optimal growth with the available therapeutic regimens is common. Changes in childhood growth and development will likely occur with the advent of new therapies that aim to reduce glucocorticoid exposure through circadian cortisol replacement or agents that reduce ACTH-driven adrenal androgens (13, 46). Our study highlights the utility of CAH-specific charts for clinical management, appraisal of growth trajectories, and the impact of new therapies.

## Data Availability

Some or all datasets generated during and/or analyzed during the current study are not publicly available but are available from the corresponding author on reasonable request.
